# A Survey-Based Study Examining Exercise in Postural Orthostatic Tachycardia Syndrome (POTS) Patients

**DOI:** 10.7759/cureus.84458

**Published:** 2025-05-20

**Authors:** Mackaleigh Levine, Devora Shapiro, Anna Hayburn, Christopher Cantrell, Robert Wilson

**Affiliations:** 1 Department of Neurology, Cleveland Clinic, Cleveland, USA; 2 Department of Social Medicine, Ohio University Heritage College of Osteopathic Medicine, Cleveland, USA; 3 Cleveland Clinic Lerner College of Medicine, Cleveland Clinic Foundation, Cleveland, USA

**Keywords:** exercise training, home-based cardiac rehab, postural orthostatic tachycardia syndrome (pots), pots-all types, pots treatment

## Abstract

Objective: To better understand why some postural orthostatic tachycardia syndrome (POTS) patients incorporate exercise regularly into their treatment and what barriers challenge others.

Background: POTS is a chronic autonomic condition in which patients experience orthostatic intolerance and abnormal tachycardia. Exercise is often recommended as a self-care modification, but many POTS patients face barriers to optimizing exercise.

Design/methods: We sent an institutional review board-approved survey via the patient e-messenger (MyChart, Epic Systems, Verona, WI) to 421 patients who participated in a shared medical appointment (SMA) by Zoom (Zoom Video Communications, San Jose, CA) within our tertiary care center between March 2022 and October 2022. The survey gathered data for the following variables: demographics, gender, body identity, symptoms that interfere with exercise, exercise tolerance, exercise prescription, attitude toward exercise, and exercise resource accessibility.

Results: Of the 421 patients messaged through MyChart, 260-286 (61.75%-67.93%) responses were formally submitted per question. A total of 221/283 (78.09%) patients have had exercise recommended as a lifestyle modification; 220/286 (76.92%) patients exercised regularly before experiencing POTS symptoms; 167/271 (61.62%) patients were unsatisfied with their exercise regimen; 111/271 (40.95%) felt like others are critical of how much they exercise; 157/267 (58.80%) patients did not feel proud of how their body looks; 116/265 (43.77%) said they are ashamed of their body; 137/265 (51.69%) patients said exercise makes them feel worse; 176/266 (66.16%) patients wanted to stop exercising due to excessive sweating; 220/267 (82.39%) patients experienced dizziness while exercising; 159/266 (59.77%) patients experienced nausea while exercising.

Conclusion: Most patients include exercise in their POTS treatment plan, and have a background in exercise before being diagnosed. However, many patients have negative self-identity and body image post diagnosis and experience symptoms while exercising that create barriers to physical activity. Managing exercise barriers for POTS patients in care delivery could improve exercise outcomes.

## Introduction

Postural orthostatic tachycardia syndrome (POTS) is a chronic autonomic condition in which patients experience orthostatic intolerance and tachycardia, especially when changing positions. The criteria for POTS in adults include a sustained increase in heart rate of 30 beats per minute when assuming an upright posture compared to a supine baseline heart rate [1]. POTS is a heterogeneous condition with variability in symptoms; patients may experience palpitations, dizziness, fatigue, headaches, hyperhidrosis, tremors, and/or GI disruptions. Recent research has demonstrated that cardiovascular deconditioning (i.e., cardiac atrophy and hypovolemia) contributes significantly to POTS and the functional disability it can cause [2]. Therefore, physical reconditioning with exercise training and volume expansion via increased salt and fluid intake is often recommended as a non-therapeutic treatment initiated early on in the initial diagnosis [2]. Exercise is often recommended to patients because it notably improves blood circulation, heart rate variability, strengthens muscles, and improves orthostatic intolerance [2-4].

However, implementing exercise as a self-care modification can be challenging for patients. While some struggle with both the physical and mental aspects of the recommended self-care modification, many patients face barriers that oftentimes limit their ability to optimize physical activity. In our tertiary care center, we idealize modifying exercise to suit the patient's current activity tolerance, while also optimizing virtual exercise opportunities that one can do at home. Then, as patients become increasingly fit, the duration and intensity of exercise should be progressively increased, and upright exercise can be gradually added as tolerated [2]. In this study, we use patient-reported outcomes to evaluate what barriers challenge POTS patients’ capacity for incorporating exercise into their treatment regimen.

## Materials and methods

Participants

The patients studied in this survey were diagnosed with POTS after undergoing a tilt table test, which a neurologist in our tertiary care center confirmed met the heart rate criteria of 30 bpm above baseline for a POTS diagnosis [1]. The diagnosing neurologist was not the same for each patient; rather, all were on the same autonomic team in a single tertiary center. Participants were 18 years or older and participated in at least one shared medical appointment (SMA) by Zoom (Zoom Video Communications, San Jose, CA) from March 2022 to October 2022. Participants were screened for these inclusion criteria via the electronic medical record (Epic Systems, Verona, WI) before being contacted for the study. Exclusion criteria included a diagnosis that was not POTS, patients under the age of 18, and patients not seen in our tertiary center. Medication management was not altered for patients who participated in the study, and written consent was obtained from all patients through the use of a study information sheet attached to the survey message.

Survey administration

An institutional review board (IRB)-approved investigator-initiated survey (Appendix) was sent to 421 patients via the electronic medical record e-messaging system (MyChart, Epic Systems, Verona, WI). An information sheet was included in the message explaining the purpose of the study, all risks, benefits, and rights as a research participant. By completing the survey, the patients acknowledged they had read the information sheet and agreed to participate in the study. The survey was intended to be comprehensive to evaluate psychological, autonomic symptoms, and social determinant barriers that could influence exercise optimization; it consisted of questions relating to the severity of POTS symptoms, patient self-identity, demographics, attitude toward exercise, and exercise resource accessibility. Responses were stored in our secure research database (REDCap, Vanderbilt University, Nashville, TN).

Statistical analysis

Of the 421 patients who received the survey through MyChart, between 260 and 286 (61.75%-67.93%) responses were submitted per question. The questions asked were multiple-choice responses, and a Likert scale (strongly agree, agree, neutral, disagree, and strongly disagree) was also used. To make the data more suitable for comprehension when analyzing the results of the questions asking the participants to rate their responses, agree and strongly agree responses were grouped, as were disagree and strongly disagree. A simple statistical analysis was performed as a comparison of percentages.

## Results

Survey results

A total of 218/261 (83.52%) patients surveyed were between 18 and 50 years old, with 242/261 (92.72%) individuals being female. A total of 221/283 (78.09%) patients had exercise recommended as a lifestyle modification by their neurologist, and 220/286 (76.92%) patients exercised regularly before experiencing POTS symptoms (Table 1).

**Table 1:** Demographics. POTS: postural orthostatic tachycardia syndrome.

Survey question	Total count (n)
Q1	n = 261
What is your age range?	
18-50	218 (83.52%)
51 or older	43 (16.47%)
Q2	n = 261
What was your sex assigned at birth?	
Male	19 (7.28%)
Female	242 (92.72%)
Q3	n = 285
Do you believe that exercise has been ordered by your provider as part of your medical treatment plan for POTS?	
Yes	221 (77.54%)
No	18 (6.31%)
Unsure	44 (15.43%)
Q4	n = 286
Did you exercise regularly before experiencing the symptoms that led to your current diagnosis?	
Yes	220 (76.92%)
No	66 (23.07%)

**Figure 1:**
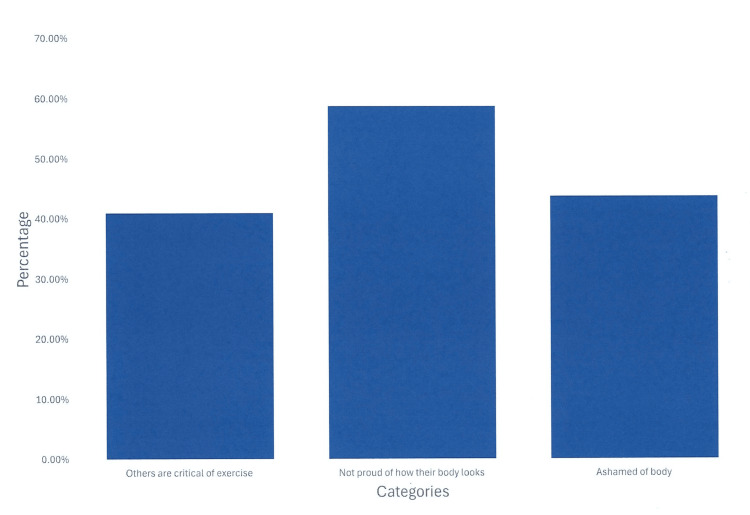
Exercise identity barriers in postural orthostatic tachycardia syndrome (POTS) patients. Examining the identity barriers of others being critical of the patients’ exercise. These were reported as a percentage of survey respondents. A total of 58.80% are not proud of how their body looks. A substantial percentage also felt ashamed of their body or felt criticized for their exercise habits.

The results related to identity barriers showed that 167/271 (61.62%) patients reported feeling unsatisfied with their exercise regimen, 111/271 (40.95%) felt that others are critical of how much they exercise, 157/267 (58.80%) patients did not felt proud of how their body looks, 116/265 (43.77%) said they are ashamed of their body, 126/267 (47.19%) reported feeling self-conscious in public because of their body, and 173/270 (64.07%) patients reported feeling self-conscious in public because, at times, they need physical assistance or accommodation (Table 2 and Figure 1).

**Table 2:** Exercise identity survey questions.

Survey question	Total count (n)
Q1	n = 271
I feel satisfied with my exercise regimen.	
Strongly disagree	65 (23.98%)
Disagree	102 (37.63%)
Neutral	61 (22.50%)
Agree	37 (13.65%)
Strongly agree	6 (2.21%)
Q2	n = 271
I feel as though others are critical of how and how much I exercise.	
Strongly disagree	36 (13.28%)
Disagree	65 (23.98%)
Neutral	59 (21.17%)
Agree	65 (23.98%)
Strongly agree	46 (16.97%)
Q3	n = 267
I feel proud of how my body looks.	
Strongly disagree	74 (27.71%)
Disagree	83 (31.08%)
Neutral	60 (22.47%)
Agree	44 (16.47%)
Strongly agree	6 (2.24%)
Q4	n = 265
How my body looks makes me feel ashamed.	
Strongly disagree	28 (10.56%)
Disagree	66 (24.90%)
Neutral	55 (20.75%)
Agree	74 (27.92%)
Strongly agree	42 (15.84%)
Q5	n = 267
I feel self-conscious in public because of my body.	
Strongly disagree	26 (9.73%)
Disagree	64 (23.97%)
Neutral	51 (19.10%)
Agree	85 (31.83%)
Strongly agree	41 (15.35%)
Q6	n = 270
I feel self-conscious in public because, at times, I need physical assistance or accommodations.	
Strongly disagree	32 (11.85%)
Disagree	33 (12.22%)
Neutral	32 (11.85%)
Agree	115 (42.59%)
Strongly agree	58 (21.48%)

**Figure 2:**
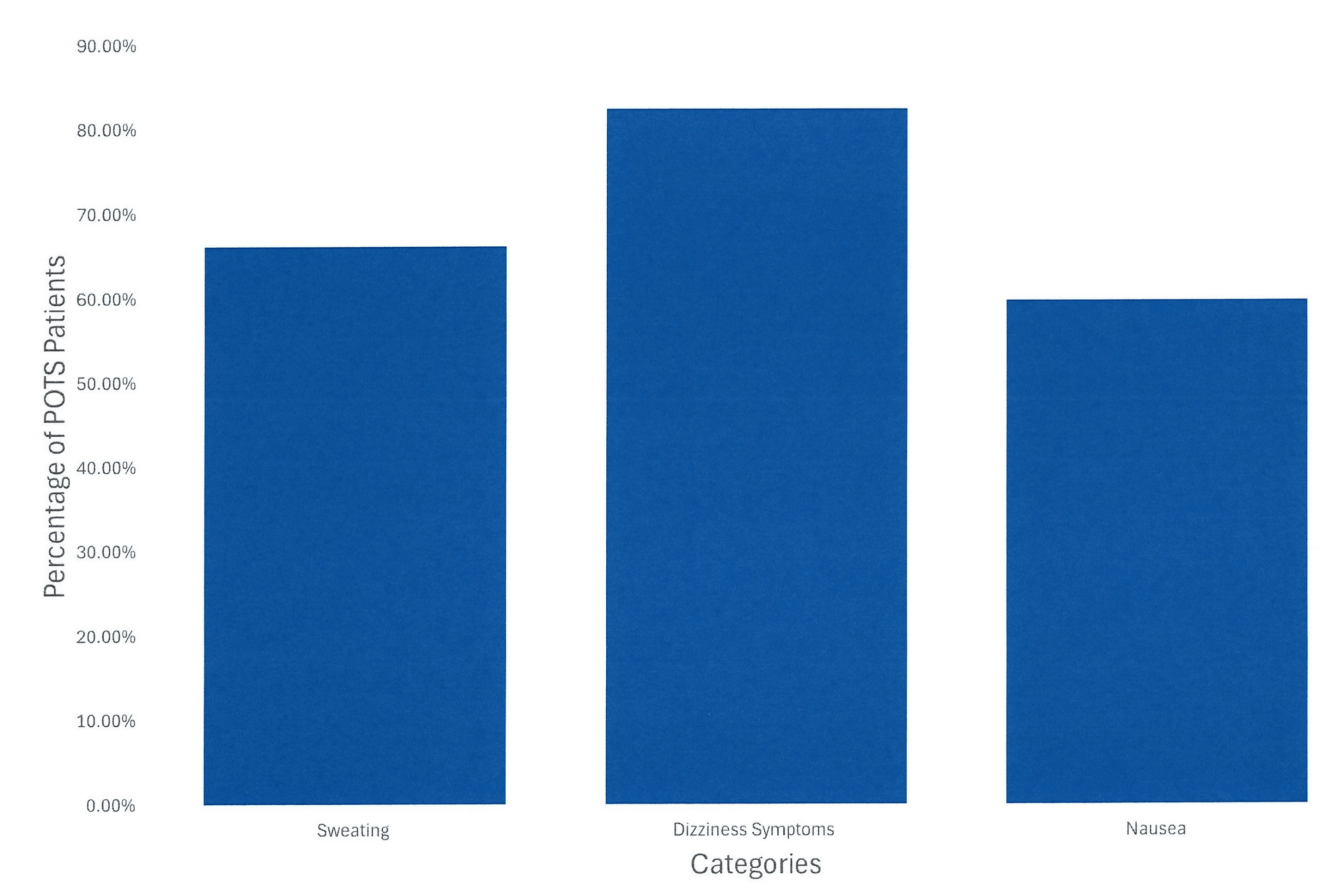
Most common symptoms in postural orthostatic tachycardia syndrome (POTS) patients during exercise. Examining the most common symptoms in POTS patients during exercise. These results were reported as a percentage. A total of 82.39% feel dizzy while they exercise. These symptoms, especially dizziness and nausea, could directly stop one from exercising.

When analyzing the symptom-related barriers our patients are most challenged by, it was concluded that 137/265 (51.69%) patients said exercise makes them feel worse, 176/266 (66.16%) patients reported wanting to stop exercising due to excessive sweating, 220/267 (82.39%) patients experienced dizziness while exercising, 159/266 (59.77%) patients experienced nausea while exercising, 140/265 (52.83%) fear they will hurt themselves during exercise due to their hypermobile joints, 96/266 (36.09%) felt the need to have a bowel movement while exercising, and 127/267 (47.56%) felt the need to urinate while exercising (Table 3 and Figure 2).

**Table 3:** Exercise symptoms survey responses.

Survey question	Total count (n)
Q1	n = 265
Exercise makes me feel worse.	
Strongly disagree	9 (3.39%)
Disagree	48 (18.11%)
Neutral	71 (26.79%)
Agree	88 (33.20%)
Strongly agree	49 (18.49%)
Q2	n = 266
Excessive sweating and/or overheating make me want to stop exercising.	
Strongly disagree	14 (5.26%)
Disagree	45 (16.91%)
Neutral	31 (11.65%)
Agree	103 (38.72%)
Strongly agree	73 (27.44%)
Q3	n = 267
Exercise makes me dizzy.	
Strongly disagree	2 (0.74%)
Disagree	19 (7.11%)
Neutral	26 (9.73%)
Agree	119 (44.56%)
Strongly agree	101 (37.82%)
Q4	n = 266
Exercise makes me feel nauseated.	
Strongly disagree	12 (4.51%)
Disagree	40 (15.03%)
Neutral	55 (20.67%)
Agree	94 (35.33%)
Strongly agree	65 (24.43%)
Q5	n = 265
My joints are unstable and/or hypermobile, and I am concerned I may hurt myself when exercising.	
Strongly disagree	42 (15.84%)
Disagree	49 (18.49%)
Neutral	34 (12.83%)
Agree	71 (26.79%)
Strongly agree	69 (26.03%)
Q6	n = 266
Do you ever feel the need to have a bowel movement when you exercise?	
Yes	96 (36.09%)
No	170 (63.90%)
Q7	n = 267
Do you feel the need to urinate during exercise?	
Yes	127 (47.56%)
No	140 (52.43%)

## Discussion

Exercise challenges

Patients are challenged by incorporating exercise as a lifestyle modification for several reasons. Our survey and clinical experience support that this is due to POTS symptom exacerbation, negative self-identity, discouraging results, feeling others are critical of their exercise, body appearance, and lack of support from friends and family.

Identity

We often see patients with chronic conditions lose their sense of self in the severity of their symptoms. Patients have expressed in relevant research and in our clinic that they feel misunderstood and dismissed by previous medical providers, healthcare teams, or support people in their lives [3].

Applicable research also suggests that many women generally have a poor body image and appearance anxiety [4-6]. In terms of exercise, those tend to feel judged, criticized, and shamed in a gym-like setting [4-6]. Leading to persistent self-criticism of their body and exercise regimen [4-6]. Therefore, exercise with a chronic condition can add more complexity and intensify negative self-identity.

Many of our patients report having a history of a fitness background before being diagnosed, which often leads to frustration related to reduced capacity for maintaining optimized movement levels. Frequently, we see patients either placed in cardiac rehab or follow a formality such as the Levine Protocol (Children's Hospital of Philadelphia (CHOP)) [7], which they may not be physically ready to endure. Many medical providers are unfamiliar with the daily behavioral and physical measures of POTS and often recommend cardiac rehab as treatment [8]. Therefore, our clinic discusses exercise to suit the needs of the patient. Emphasizing the methods of gradual exercise progression, optimizing at-home activities, and encouraging the patient to partake in activity at their comfort level.

Psychological barriers

Existing literature has identified mental health-related barriers to engagement in exercise for people with chronic pain/illness, including low motivation due to anxiety or depression, kinesiophobia, fear of increased symptoms, fear of injury, poor self-image, and perceived lack of understanding regarding proper exercise techniques [9,10]. Given that people with POTS have been found to have a higher rate of somatic focus and fear of symptoms [11], they may be more likely to interpret increased symptoms during exercise (e.g., palpitations and shortness of breath) as harmful, which could lead to avoidance behaviors.

A recent study conducted by our institution [12] found that POTS patients with an anxiety disorder had a significantly higher likelihood of treatment changes for their autonomic symptoms. Notably, common symptoms of anxiety include rapid heartbeat, excessive sweating, shortness of breath, dizziness, unexplained aches and pains, difficulty sleeping, trouble concentrating, and or irritability. POTS is not caused by anxiety; however, it should be noted that one can have anxiety alongside POTS. In some cases, low-dose propranolol is used to manage both POTS and anxiety symptoms [7]. Having baseline physiological anxiety symptoms from POTS combined with an anxiety disorder could potentially contribute to worse symptoms during exercise compared to an individual without these challenges.

More than half of the participants in the current survey indicated troubles with negative body image, and almost half indicated they had received criticism from others about exercise. Internalized shame and guilt are associated with exercise avoidance. Although it is recommended for patients to view exercise as rehabilitation and start slow to reduce the likelihood of symptom exacerbation, they may still struggle with negative thoughts such as not doing enough, not making progress, or comparison to others.

Symptom barriers

Heat sensitivity, or excessive sweating, can limit exercise and should be explained to the patient in POTS education. Understanding the patient’s experience of heat sensitivity, sweating, and fatigue outside of exercise intolerance can give guidance toward a feasible, structured exercise program that is patient-centered.

Cognitive symptoms are often under-reported and may have significance for exercise programs to the degree of understanding, execution, and sustainability. Careful review of each patient’s symptoms and including them in the decision-making process can promote the formation of patient-centered exercise programs designed with the intention of gradual advancement.

Comorbidities

Adrenaline in the sympathetic state can cause POTS patients to experience exacerbated symptoms [7]. Being self-aware of symptoms is essential when identifying sympathetic overdrive to avoid greater disruption of neurological signaling.

In our clinic, we often see POTS patients experience pain through common comorbidities like fibromyalgia (FM), headaches, and migraines. FM is a pain-related comorbidity [13]. Anecdotal reports suggest that patients with FM limit their exercise due to pain and fatigue [13-15]. Although there are few empirical studies published regarding exercise in this patient population, it may be that predictors of these behavioral changes, such as exercise avoidance in patients with FM, differ from those of healthy individuals [13].

Another chronic condition POTS patients are challenged by is small fiber neuropathy (SFN). SFN is best described as pain, numbness, or tingling in the hands or feet. Sometimes SFN can also cause excessive sweating. Therefore, exercise can be difficult for POTS patients who have SFN because these symptoms limit exercise tolerance.

Ehlers-Danlos syndrome is a demographic of POTS patients that needs more exploration. Approximately 20%-30% of the POTS population also meets the diagnostic criteria for EDS [16]. EDS patients have challenges with joint pain, joint disorders, and hypermobility that can pose difficulty during exercise. Further understanding of a patient-centered approach to exercise for POTS patients with EDS, including ideal tolerance, consistency, and outcomes, is of further merit and investigation.

Exercise can be a precipitant to migraines, headaches, and neck pain. Migraines and headaches, associated symptoms of fatigue, nausea, malaise, photophobia, and phonophobia, can impact the ability and willingness to participate in exercise. Understanding the patient’s headache history and providing treatment can help improve the involvement of an exercise program [17].

Exercise can also be a preventive measure for migraines. Understanding the patient's perspective on how headaches, migraines, and neck pain factor into exercise tolerance and exercise achievement is important in the delivery of care for a POTS patient. Since migraine and headache can be associated with global impairment, the treatment of these conditions can lead to the potential for more opportunities in exercise engagement [17,18].

Limitations

This study had limitations, which included a select population from our tertiary care center. Therefore, our ability to generalize the results is limited. Our survey generated patient-reported outcomes that reflect our patients’ experiences at a single tertiary center; while this provides an important quantification, such data can be subject to bias.

The sample likely reflects a more engaged or severe population (those attending Zoom SMAs and having access to MyChart). Patients who responded may differ from non-respondents in motivation, symptoms, or attitudes toward exercise. Grouping Likert responses (“agree” with “strongly agree”) may also obscure nuance.

The study is cross-sectional and cannot infer causality or change over time. Surveys were not validated scales, but rather IRB-approved investigator-initiated. The overwhelmingly female sample limits conclusions for other populations. Symptom severity during exercise was not matched to objective metrics (e.g., heart rate, blood pressure, and tilt table test outcomes), limiting clinical interpretability.

Examining exercise barriers for POTS patients through a survey is the best option we had to evaluate the general perspective due to the subjective nature of the symptoms in our patient population. Investigating the specific challenges presented by each symptom during exercise could be examined in future studies.

## Conclusions

Most patients have exercise recommended to them in their POTS treatment plan, but they also have a background in exercise before being diagnosed. A considerable number of patients have a negative self-identity, body image, and limiting symptoms post diagnosis that create barriers to physical activity. Many women, which makes up a large percentage of our patient population, often struggle with the physical and psychological aspects of exercise without having chronic conditions. Therefore, when recommending exercise to a patient with POTS, the importance of the conversation in clinical care delivery is substantial for guiding the patient's exercise regimen.

Based on the survey results, understanding the patients' symptomatology, self-identity, psychological background, and co-existing comorbidities will help lead the exercise discussion. Knowing the identity barriers may help medical professionals design exercise regimes beyond physical symptoms. Understanding the emotional and psychological needs of the patient can help guide a program concerning how the patient views themself and their illness. Specific exercises cannot be generally concluded, as each patient is unique. Further research to evaluate the patient-specific barriers may result in ideal forms of exercise for the patient and may also help other people with other autonomic dysfunction. Collaboration between the patient and medical providers to customize the exercise approach to the individual, emphasizing the importance of respecting their boundaries, understanding the symptomatic barriers, and optimizing at-home exercise programs due to poor body image and identity concerns, could improve overall exercise outcomes. Future research to investigate patient-centered exercise programs can give insights into outcomes for POTS recovery and treatment using exercise.

## Figures and Tables

**Figure 3:**
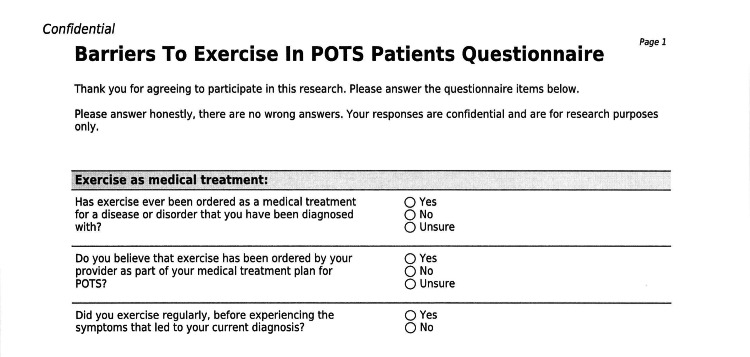
Barriers to exercise in POTS Patients Questionnaire. POTS: postural orthostatic tachycardia syndrome.

**Figure 4:**
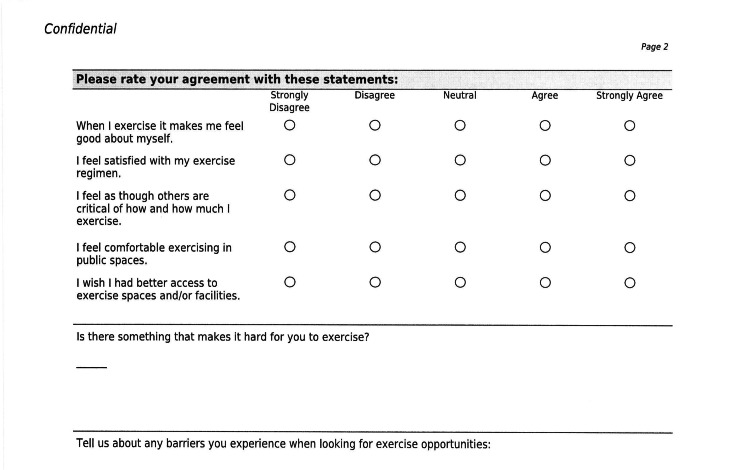
Exercise identity questions.

**Figure 5:**
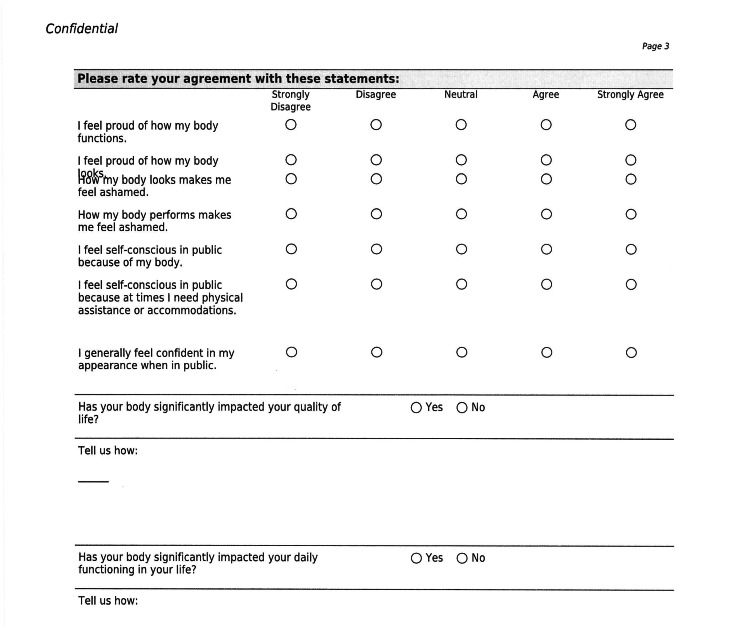
Exercise identity questions.

**Figure 6:**
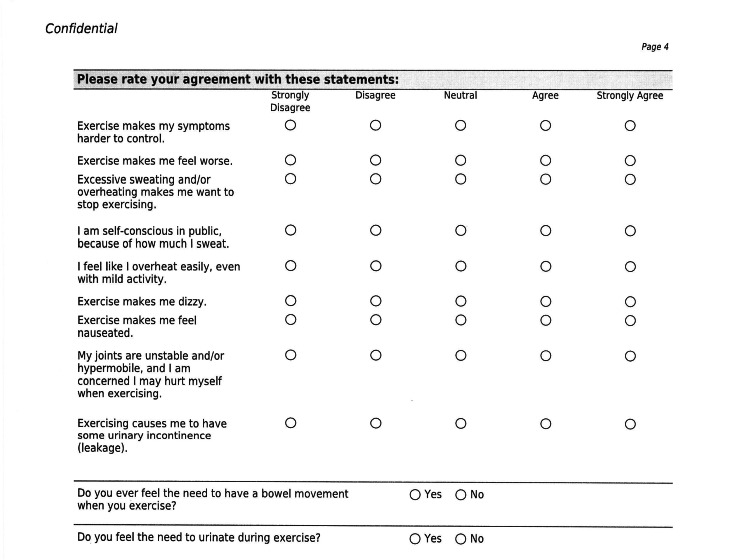
Exercise symptoms questions.

**Figure 7:**
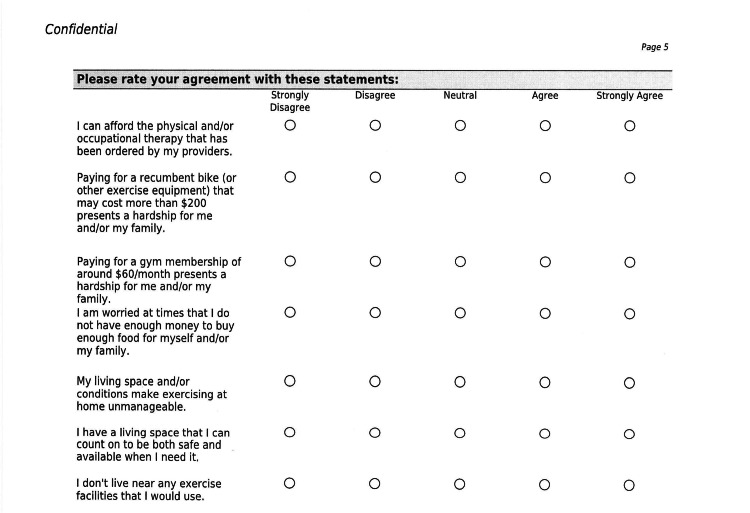
Exercise accessibility questions.

**Figure 8:**
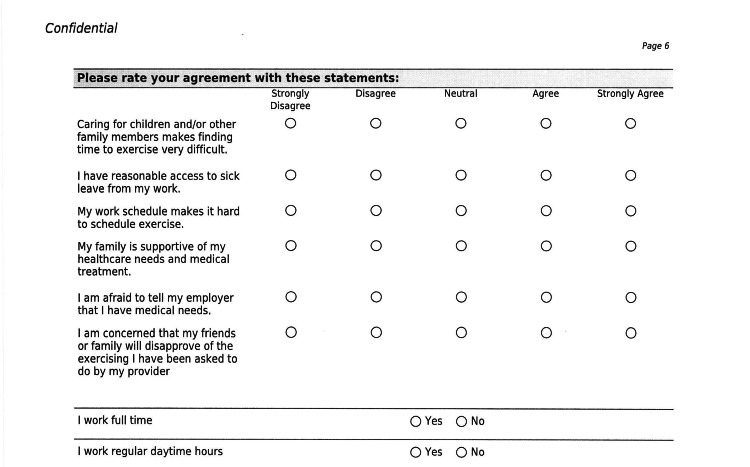
Exercise support questions.
